# Radiographic Evaluation in Implant Patients: A Review

**DOI:** 10.7759/cureus.54783

**Published:** 2024-02-23

**Authors:** Shrishti S Salian, Chitrika P Subhadarsanee, Ruchita T Patil, Prasad V Dhadse

**Affiliations:** 1 Department of Periodontics and Implantology, Sharad Pawar Dental College & Hospital, Datta Meghe Institute of Higher Education & Research, Wardha, IND

**Keywords:** lateral cephalograph, opg, iopa, denscan, cbct, radiographic evaluation, implant

## Abstract

Diagnostic imaging is crucial in assessing dental implant patients. The height, width, and shape of the bone are precisely depicted and measured by these tests, which help pinpoint the locations of significant anatomical structures adjacent to the implant placement sites. The type of implant to be utilized, the positioning of the remaining dentition, and the degree to which bone quality or quantity is in question all play a role in determining the radiologic approach that is most suited for a given patient. This review is an update on the current knowledge in the field of radiographic evaluation in implant placement. Considering the radiation exposure and the expense of each test, it is important to carefully determine whether pre-implant imaging is acceptable in each situation. Although multislice computed tomography is the gold standard from the authors’ perspective, not every implant situation can justify such a test.

## Introduction and background

The first step in creating a functional and visually pleasing implant prosthesis is choosing the right implant site and the best implant for that site. Diagnostic imaging is crucial in assessing dental implant patients. The bone’s height, width, and shape are precisely depicted and measured by these tests, which help pinpoint the locations of significant anatomical structures adjacent to the implant placement sites. The implant type to be utilized, the remaining dentition positioning, and the degree to which bone quantity or quality is in question all play a role in determining the radiologic approach that is most suited for a given patient. A complete radiographic evaluation is essential for assessing these aspects, letting patients know their chances of a good recovery, appraising and guiding implant placement before surgery, and assessing the prognosis of the implant after surgery [1].

Nowadays, imaging options include tomography, computed tomography (CT), intra-oral radiography, cone beam volumetric tomography, traditional extra-oral radiography, and magnetic resonance imaging. The suitability of each of the imaging options will be examined, and information will also be provided on interpretation [1].

## Review

Objectives of imaging

The goals of diagnostic imaging are affected by several variables, including the quantity and type of information needed and the time of the procedure. These factors are taken into account while choosing the imaging modality and timing, which can be broken down into two parts [1].

Pre-Surgical Imaging (Phase 1)

A pre-surgical radiographic examination is used to assess the quality and quantity of existing bone, the bone’s angulation, the location of prospective implant sites, and to rule out any pathology. Radiation dosage should be taken into consideration while choosing the best radiographic modality in both dental and medical radiology. Always follow the “as low as reasonably achievable” (ALARA) approach, which emphasizes that the diagnostic imaging method should expose the patient to the least amount of radiation possible. The radiation dose should not, however, jeopardize patient care or treatment planning. According to recommendations from the American Academy of Oral and Maxillofacial Radiology, every implant site evaluation should be conducted using a three-dimensional imaging method like conventional or computerized tomography. The main objective of this treatment phase is to design and implement a patient treatment plan that enables the restoration of the patient’s function and appearance through the exact and deliberate placement of dental implants [2].

Intra-Operative and Surgical Implant Imaging (Phase 2)

The goal of phase two, also known as surgical and intra-operative implant imaging, is to support the patient’s surgical and prosthetic interventions. This imaging phase’s objectives include evaluating the surgical sites before, during, and after the treatment, assisting with dental implant placement and orientation, evaluating how well the implants are healing and integrating, and confirming that the abutment and prosthesis were correctly constructed [2].

Periapical radiography: Images of a specific area of the mandibular or maxillary alveolus are captured by periapical radiography. To take a periapical radiograph, place the film in the mouth so that it is parallel to the body of the alveolus and point the X-ray machine’s center beam at the desired area. As a result, the alveolus can be seen from the side. Due to the intrinsic distortion of the resulting image, using the bisecting angle approach for periapical radiography should be avoided. Only a portion of the structures being scanned using the bisecting angle technique is dimensionally accurate because the technique uses a geometric trick to create the image. The long cone paralleling approach is recommended for capturing periapical radiographs because there is no superimposition of the zygoma over the upper molar region and a real relationship between the bone height and neighboring teeth is seen [3].

The advantage is that these films are most typically used for single-tooth implants during the pre-prosthetic stage in places with ample bone width. They are perfect for documenting and assessing any potential peri-implant bone resorption during follow-up. Additionally, the small amount of radiation is also one of the advantages.

The limitation is that the image is of little use in determining the quantity because it is magnified, subject to distortion, and lacks representation of the third dimension of bone width. Moreover, it has little use in calculating mineralization or bone density [1].

Occlusal radiography: The central X-ray is perpendicular to the film for the mandibular image and oblique (45°) to the film for the maxillary image when taking planar radiographs or occlusal radiographs taken inside the mouth.

The advantages are that periapical radiographs are unable to offer any cross-sectional information, and occlusal radiographs are occasionally used to assess the buccolingual dimensions of the mandibular alveolar ridge.

The limitation is that the maxillary occlusal radiographs are naturally obliquely distorted, and they are of less value in implant dentistry in measuring the level of mineralization of the implant site or the geometry. Additionally, it contrasts the breadth of the crest of the bone, where diagnostic information is most needed, with the widest part of the bone [4].

Lateral cephalometric radiographs: Oriented planar head radiographs are another name for cephalometric radiographs. A cephalometer, which fixes the physical location of the skull using projections into the external auditory canal, is used to position the skull in relation to the X-ray machine and the image receptor. The image is magnified by 7-12% due to the geometry of cephalometric imaging devices.

The advantage is that the lingual plate’s link to the patient’s skeletal structure and the geometry of the anterior alveolus are both seen on the lateral cephalometric radiograph, which is helpful.

The limitation is that this method only shows a cross-sectional image of the alveolus where the central X-ray device beams are tangent to the alveolus, which is not helpful for assessing bone quality. Other disadvantages include image magnification and superimposition [4].

Panoramic radiography: The lower half of the maxillary sinuses, the body of the mandible, and the maxilla are all included in a single image created using the curved plane tomographic radiographic technique known as panoramic radiography. This radiography procedure creates a picture of a jaw portion with varying thickness and magnification.

The advantage is that identifying opposing landmarks is simple. One can gauge the bone’s initial vertical height. It is useful in making preliminary estimations of cortical boundaries and crestal alveolar bone. The process is completed quickly, conveniently, and easily. The jaws’ gross anatomy and any associated pathologic abnormalities can be assessed. Throughout the panoramic X-ray examination, the patient has diagnostic templates. These templates have 5-mm ball bearings or wires incorporated along the dental arch’s curve, allowing the doctor to choose the radiograph’s degree of magnification [1].

The limitation is that because different sections of the radiograph exhibit distinct distortions, it can be challenging to determine the density and shape of hard tissues with accuracy. Clinicians cannot determine the alveolar ridge’s slope or the buccolingual cross-sectional dimension from a panoramic image. Mesiodistal distance measurements can be quite inaccurate because of poor patient posture and/or individual variances in jaw curvature. In terms of illustrating the spatial linkages between the structures and the dimensional quantification of the implant site, it is only marginally useful for highlighting important structures.

Zonography: A cross-sectional image of the jaws can now be created using a modified version of the panoramic X-ray system. These devices position the subject and use limited-angle linear tomography (zonography). The tomographic layer is around 5 mm thick. With the aid of this technique, it is possible to measure the implant site’s geometry and comprehend the spatial relationship between the crucial elements and the implant site. The effectiveness of this approach for specific sites is constrained by the thick tomographic layers and neighboring structures that are blurred and superimposed on the image, particularly in the anterior regions where the shape of the alveolus varies quickly [4].

Tomography: For pre- and post-implant evaluation, tomographic units can create cross-sectional slices of the jaws that are as thin as 1 mm. In the case of digital images, measurements can be taken using a measuring program after calibration because the images are produced at a consistent, known magnification. Measurements can be taken directly from the images using a particular ruler fitted with the appropriate scale. Tomography requires precise expertise since superimposing features outside of the plane of focus causes the image to become noticeably blurry, making it exceedingly challenging to read them. Several movement patterns, such as linear, spiral, or hypocycloidal, have been tried to lessen the blurring of artifacts and provide a crisper and more useful image. Utilizing this method when there are several implant locations is challenging. Images that are hard to understand could be the consequence of anatomical differences [5].

CT: Sir Godfrey Hounsfield created CT, which was introduced to the imaging community in 1972. By the early 1980s, complicated tomography had been largely supplanted by CT scanning, thanks to the introduction of the first CT scanners in medical imaging departments in the middle of the 1970s. Unlike high-contrast skeletal structures, soft tissues, especially the brain, were the focus of CT’s development. Similar to conventional tomography, conventional (incremental) CT creates sectional images using X-rays. High-resolution pictures can be produced by scanning in an axial plane with thin sections and contiguous or overlapping scans (maxilla: 20-30 axial slices; mandible: 30-35 axial slices, 1.5 mm thick and 1 mm apart with 0.5 mm overlapping) [5].

The planning of dental implant surgery using CT has been well described. Utilizing CT scanning is highly recommended since it allows for an accurate pre-operative analysis of the available bone volume and supports the selection of the appropriate location, angulation, quantity, and length of the intended implants. The soft tissues can be partially seen with this modality, which also provides a high-density resolution. The axial, panoramic, and cross-sectional images from the reformatted CT images are all cross-referenced to one another, enabling quick correlation of the various views. Compared to periapical, panoramic, and hypocycloidal radiography, CT offers a significantly more accurate assessment of the location of the mandibular canal. Compared to panoramic radiographs, the anterior mandibular buccal depression is easier to spot on a CT scan. The only reliable method for determining the amount of bone behind the maxillary sinuses is computed tomographic scans with reformatted pictures. Anatomical structures, like the cortical bone, have clearer margins in computed tomographic pictures than in dosimetric tomographic images. These allegedly distinct borders result from the calculated linear attenuation for a voxel, which is the weighted average of all tissues. This phenomenon, known as partial volume averaging, may lead to inaccurate representations of bone thickness and compromise the accuracy of measurements [6].

The advantage is that by reformatting the image data to provide tangential and cross-sectional tomographic views of the implant site, CT offers a distinctive method of imaging evaluation of potential surgical or implant sites. Reformatted images produced by modern CT scanners have a geometric resolution comparable to planar imaging with a section thickness of 1 pixel (0.25 mm) and an in-plane resolution of 1 pixel multiplied by the scan spacing (0.5-1.5 mm). The absolute and quantitative density of the structures in the image can be used to differentiate between the various tissues in the region and evaluate the quality of the bone. Several techniques known as “Dentascan imaging” were developed as a result of the advantages and delivery limitations of this type of imaging [7].

Dentascan: For patients with cysts, tumors, distractions, and cases of fractures in either the mandibular or maxillary arch, Dentascan examination is a specialized type of CT study performed on a standard CT scanner. It aids in assessing factors such as the accuracy of root canal obturation, jaw growth, stages of tooth development, and dental implants.

Advanced computer programs employ Dentascan to assess an X-ray study by generating detailed two-dimensional and three-dimensional images, which enable nearly accurate diagnosis and surgical planning well in advance of the procedure. Typical dental X-rays are simply two-dimensional, displaying only the position of the teeth and the height of the bone. These X-rays are frequently distorted and unable to show the thickness of the jawbone; in contrast, a dental CT scan is distortion free. It offers cross-sectional views of the jaws in three dimensions and depicts the true bone composition. The life-sized photographs make it possible to gauge the quantity and density of bone precisely.

The advantages are the determination of bone thickness and height, identification of pathology in both soft and hard tissues, recognition of anatomical landmarks, such as the interdental canal, and the ability to assess the critical qualitative parameters required for implant placement.

The limitations are that images might not be their full size, and magnification compensation could be necessary. The imaging computer or workstation must be used to determine the quality of the bone. Hard copy Dentascan images only offer a small selection of the diagnostic gray scale of the study, and because all cross-sectional images are taken perpendicular to the axial imaging plane, tilting the patient’s head during the examination is crucial [8].

Cone beam CT (CBCT): A three-dimensional cube or voxel is equivalent to each two-dimensional pixel in two-dimensional imaging. The total X-ray absorption across each voxel is calculated for each pixel. Low-dose CBCT, which uses a cone-shaped X-ray beam rather than the flat fan-shaped beam used in normal CT, has been able to get around this two-dimensional restriction. The total effective dosage is between two and eight panoramic radiographs or 0.035-0.10 mSv. Greater resolution is achieved because individual voxels are substantially smaller than those found in traditional CT scans. Examples of such machines include NewTom DVT 9000 (Quantitative Radiology, Verona, Italy), i-CAT (Imaging Sciences International, Hatfield, United States), and 3D Accuitomo (J. Morita, Kyoto, Japan) [6].

These imaging techniques might work well on patients who are missing all of their teeth or who are being considered for the implantation of numerous implants. The axial computed tomographic image slices are obtained with the jaws set so that they are parallel to the occlusal plane. There are roughly 30 axial image slices per jaw from these thin (1-2 mm) and overlapping axial pictures. Using a computer-based technique known as multiplanar reformatting, these sequential axial images’ image data can be post-processed to produce a variety of two-dimensional images in various planes.

The pictures of oral structures produced by CBCT were superior to those produced by multidetector CT (MDCT) 33, as reported in a recent study that compared CBCT with the most advanced medical multislice CT, the multidetector row helical computer tomography. The future of dental implant treatment planning is CBCT due to the reduced radiation dose and more convenient upright scanning position.

The advantage is that computed tomographic scans frequently show the continuity of the cortical plates, the quantity of residual bone in the mandible and maxilla, the relative positioning of nearby significant structures, and the shape of the soft tissues covering the osseous structures. It has been demonstrated that pictures that have been reformatted from CBCT data have measurement precision on par with MDCT data. These reformations can assess the interior density and are helpful in planning augmentation treatments like a sinus lift. A depiction of the general anatomy of the desired implant location can be seen in three dimensions.

The disadvantage is that striking artifacts brought on by metallic restorations can be avoided by positioning the jaws so that the acquired axial scans are parallel to the occlusal plane [9].

Interactive software: A variety of software programs have been created to provide pre-surgical computer modeling of implant orientation and placement. Both “CT (SURGEPLAN)” and “Reformatted computed tomography (Dentascan, SimPlant)” can use the software. There are also other software programs like “Procera software (Nobel Biocare, Sweden V implant (CyberMed), Seoul, South Korea).” These programs offer an interactive platform that enables investigation of the quantity, quality, and morphology of the bone at potential implant locations [10].

Imaging stents: An imaging stent that aids in connecting radiographic images and their information to a particular anatomic position or potential surgical site can improve pre-surgical imaging. Radiographic spheres or rods kept within acrylic stents can be used to identify the implant sites. The insertion angle of the guide bar and, ultimately, the angle of the implant, can be oriented using these as a surgical guide. Only non-metallic radio-opaque markers, such as guttapercha, resins, and composites, should be used since metallic markers cause artifacts in CT [10].

Discussion

While there are numerous ways to scan the implant site, the appropriate method should be chosen based on the circumstances and the clinician’s assessment of how best to interpret the obtained image. Because each pre-implant imaging examination is costly and involves radiation, it is important to carefully examine which type of imaging is appropriate for each patient. While the authors believe multislice CT to be the top standard, not all implant cases need this kind of testing. Pre-implant imaging is a promising application of cone beam volumetric tomography. The radiation dosage the patient will get should not be the only factor considered by the practitioner when selecting suitable imaging. [11].

Hung et al. [12] investigated the current clinical applications and diagnostic performance of artificial intelligence in dental and maxillofacial radiology. Artificial intelligence applications in radiology could be another future investigation method for implant patients. Virtual monoenergetic images at high virtual monoenergetic energies utilizing dual-energy CT datasets can eliminate metal artifacts [13].

Solution-processed materials have been developed recently to advance next-generation X-ray imaging systems with low cost, high sensitivity, and flexibility. Perovskites, in particular, have gained attention as attractive materials for radiation detection, luminescence displays, and photovoltaic systems due to their variable bandgap, strong photoluminescence quantum yields, narrow emission, and high charge-carrier mobility [14]. As an X-ray scintillating screen for high-resolution radiography, room-temperature synthesized CsPbBr3 nanosheets can be put together to form a homogeneous, thick layer [14]. Digital radiography (DR) techniques are another technique to assess for non-destructive testing on aircraft components. Their analysis suggests that, although DR requires shorter exposure times than registered radiography, both techniques offer comparable imaging capabilities. Takahashi et al. [15] employed the transfer learning technique on five convolutional neural network (CNN) architectures to categorize four distinct implant systems, exhibiting a 90%+ accuracy rate across all models. It is possible to detect dental implants using the YOLO network, which is specialized in object detection. Using 11,980 panoramic and periapical radiographs, Lee et al. [16] employed an automated deep CNN (DCNN) model to categorize six distinct implants. According to the findings, the automated DCNN performed better than the majority of the dental specialists who took part, including residents and periodontists. Based on dental radiography pictures, CNN has shown a high degree of effectiveness in categorizing similar shapes of various implant system kinds. Given the wide range of dental implant types on the market, more research and development may be able to categorize various dental implant systems, which will help physicians diagnose and treat implant failures by providing important information.

## Conclusions

There is very little to be gained by opting for pre-implant imaging, where the dose is very low if the result is compromised because of a lack of reliable information. The risk-benefit ratio should be determined on an individual basis to maximize success. Due to their ability to provide the best radiographic survey at a low dosage, panoramic radiography and CBCT are widely used as the standard radiographic examinations for the treatment planning of implant patients. Periapical radiographs are meant to fill in the gaps or clarify specifics left by the panoramic radiograph and CBCT.
